# Spatial Analysis of Flood Risk, Neighborhood Characteristics, and Chronic Health Conditions in North Carolina

**DOI:** 10.1029/2024GH001295

**Published:** 2026-02-02

**Authors:** S. E. Ulrich, M. M. Sugg, S. M. Hatcher, J. D. Runkle

**Affiliations:** ^1^ Department of Geography and Planning Appalachian State University Boone NC USA; ^2^ Occupational and Environmental Epidemiology Branch Division of Public Health North Carolina Department of Health and Human Services Raleigh NC USA; ^3^ North Carolina Institute for Climate Studies North Carolina State University Asheville NC USA

**Keywords:** local indicators of spatial autocorrelation, index of concentration of extremes, flooding, spatial clustering

## Abstract

Climate change will continue to increase the frequency and intensity of flood events in North Carolina for the foreseeable future. The extreme flooding in Western North Carolina caused by Tropical Storm Helene in September of 2024 is a recent and devastating example of this trend. Communities of color and low‐income populations are more likely to reside in flood‐prone areas due to structural factors, including residential racial segregation and economic inequality. As such, the adverse health and financial consequences of flood exposure overburden historically marginalized communities, which may have a more limited adaptive capacity to anticipate, respond to, and recover from flood events. Exposure to severe flooding further exacerbates chronic health conditions by impeding access to vital healthcare infrastructure and services. This study examines the spatial patterning of coastal and inland flood risk, neighborhood‐level structural determinants (i.e., racial and economic inequality), and flood‐sensitive health conditions in North Carolina using bivariate local indicators of spatial autocorrelation (LISA) statistics. High‐high clusters capture areas where neighborhoods with high racial or economic inequality surround elevated flood risks. These clusters are distinguished by select sociodemographic characteristics and concentrated in the eastern coastal and western mountainous regions of North Carolina. Cluster locations are priority areas for targeted resource allocation and interventions that strengthen the adaptive capacity of these communities in the context of climate change.

## Plain Language Summary

Climate change is increasing the frequency and severity of floods in North Carolina, tragically demonstrated by Hurricane Helene, which caused a rare 1‐in‐1000‐year flood throughout the state's western region. A community's ability to prepare for, respond to, and recover from floods depends heavily on access to financial and material resources. Communities of color and low‐income populations are more likely to live in flood‐prone areas, making them especially vulnerable to the health and economic impacts of flooding. Flooding can also worsen chronic health conditions by disrupting access to healthcare services. This study maps flood risks across the state alongside neighborhood characteristics like racial and economic inequality and flood‐sensitive health issues. The study identifies areas where high levels of inequality overlap with severe flood risks. Using spatial statistical analysis, these high‐risk clusters are found mainly in North Carolina's eastern coastal and western mountainous regions. These areas should be prioritized for resources and programs to help communities better adapt to the challenges of climate change.

## Key Points

- Spatial clustering of both flood exposure and socioeconomic inequity reveals dual, compounding challenges in the western and southeastern regions of NC
- The integration of inland flood metrics reveals areas of elevated flood risk in Western NC that were affected by Tropical Storm Helene
- High‐high clusters of flood risk and chronic health conditions (asthma, COPD, diabetes, depression, kidney disease) identify priority areas for public health interventions

## 1 Introduction

Climate‐related changes in precipitation patterns coupled with rapid floodplain development have significantly increased inland flood exposure across the United States (Pal et al., 2023; Tate et al., 2021; Wobus et al., 2021). The severity of this risk was demonstrated by the devastation caused by Tropical Storm Helene in Western North Carolina in September 2024, where over 30 inches of rain were recorded in some areas. The storm shattered North Carolina's century‐old record for flood‐related fatalities, resulting in 104 deaths statewide, including 42 in Buncombe County (NC DHHS, 2024). Beyond the tragic loss of life, Helene exposed critical vulnerabilities in healthcare systems, infrastructure, and community resilience (Crumpler, 2024; North Carolina Office of State Budget and Management, 2024). Additional impacts included widespread healthcare disruptions, shortages of essential medical supplies, and increased exposure to environmental hazards such as contaminated water and swarms of displaced insect populations (OSBM, 2024). Over the past decade, North Carolina has experienced increased heavy precipitation, more frequent and intense severe weather events, and inland flooding, further exacerbated by sea level rise (North Carolina Department of Environmental Quality, 2024). These trends, coupled with escalating flood severity as seen during Helene, disproportionately impact vulnerable populations and worsen existing inequalities through physical destruction, financial hardship, and adverse health outcomes (Thomson et al., 2021).

The extent of a flood event's impact on health, property, and infrastructure is shaped not only by its severity and frequency but also by individual, structural, and geographic factors (Bergstrant et al., 2015; Lowe et al., 2013; Mason et al., 2021; Ziegelaar & Kuleshov, 2022). Material and financial resources significantly influence communities' capacity to anticipate, respond to, and recover from floods (Mason et al., 2021; Qiang, 2019; Srikuta et al., 2015; Tate et al., 2021). Structural racism, manifested through systemic policies, practices, and norms, has historically restricted access to these resources for marginalized communities, compounding vulnerabilities to environmental hazards such as extreme heat and flooding (Bailey et al., 2017, 2021; Braveman et al., 2022; Breaky et al., 2024; Williams et al., 2019). Historical practices like redlining and racial residential segregation have placed communities of color in areas of higher flood risk while limiting access to economic opportunities, safe housing, and healthcare (Ndugga et al., 2024). Residents of flood‐prone areas in the U.S. are disproportionately racial and ethnic minorities and low‐income populations (Aggarwal et al., 2023; Lee & Jung, 2014; Qiang, 2019; Rolfe et al., 2020; Tate et al., 2021), with climate projections suggesting that Black communities in the U.S. will face the most severe increases in flood risk due to climate change (Wing et al., 2022). These inequities exacerbate the adverse health impacts of floods and reduce the capacity of affected communities to recover. Older adults, people with chronic disease, young children and people with young children, and people with one or more disabilities also face an elevated risk of adverse outcomes related to flood exposure (Lee et al., 2020; Lowe et al., 2013; Mason et al., 2021; Srikuta et al., 2015; Wu et al., 2015). Flood exposure exacerbates existing health conditions, including respiratory diseases (e.g., asthma, COPD), chronic diseases (e.g., diabetes, kidney disease), and mental health outcomes such as depression (Aggarwal et al., 2023; Bei et al., 2013; Bich et al., 2011; Du et al., 2010; Fernandez et al., 2015; Lee et al., 2020; Lowe et al., 2013; Milojevic et al., 2017; Saulnier et al., 2018; Twiddy et al., 2022). Breakdowns in infrastructure and disruptions to essential services during and after flood events further compromise health, as evacuations, displacement, and medical facility closures disrupt medication adherence, treatment continuity, and healthcare access (Aggarwal et al., 2023).

A significant limitation in studies of flooding, neighborhood characteristics, and health outcomes is a lack of assessment of the risk of exposure to both pluvial and fluvial inland flooding hazards (Tate et al., 2021), despite evidence that marginalized communities experience disproportionately higher exposure to inland flood events (Qiang, 2019; Rolfe et al., 2020). Pluvial flooding results from extreme rainfall independent of nearby water bodies, whereas fluvial flooding occurs when rivers or streams overflow their banks. Most studies on flood‐related health outcomes focus on major coastal disasters, leaving inland pluvial and fluvial flooding understudied (Fernandez et al., 2015). Additionally, research on inland flooding has also been constrained by the historical absence of spatially contiguous floodplain data, including gaps in federally designated flood maps.

The objective of this study is to identify spatial clusters of pluvial and fluvial flood risk in relation to community‐level structural and health vulnerabilities in North Carolina. This approach highlights geographic areas where elevated flood risk overlaps with social inequities and chronic health burdens, identifying populations facing compounded risks from flood‐related disasters and informing targeted strategies for climate resilience and public health planning. By integrating inland flood metrics, this study addresses a critical gap in flood‐health research for regions like western North Carolina, where the recent devastation from Tropical Storm Helene underscored the urgency of identifying vulnerable communities.

## 2 Data

### 2.1 North Carolina Flood Risk Data

The physiographic regions, major rivers, county boundaries, and major cities of the study area, North Carolina (NC), are shown in Figure S1 in Supporting Information S1. We assessed flood hazards and their associated risk for infrastructure across NC using the First Street Foundation's Flood Model (FSF‐FM) data set, which uses pluvial, fluvial, and coastal flood modeling to estimate overall flood risk for each property in the U.S. FSF‐FM risk estimates capture a greater extent of flood risk compared to federal flood maps, which do not account for pluvial flooding and vary in spatial quality (First Street, 2023; Qiang et al., 2017; Tate et al., 2021; Wing et al., 2017). The FSF‐FM assigns each property within a census tract a FloodFactor(™) score of 1–10 to indicate minimal to extreme flood risk, respectively. The FSF‐FM defines a FloodFactor(™) of 1 as minimal risk, 2 as minor risk, 3–4 as moderate risk, 5–6 as major risk, 7–8 as severe risk, and 9–10 as extreme risk (First Street, 2023). Based on these categorizations, we calculated the proportion of low (FF ≤ 2), moderate (FF 3–6), and high (FF ≥ 7) risk properties within each census tract in North Carolina using the July 2023 iteration of the FSF‐FM.

### 2.2 Index of Concentration at the Extremes

The Index of Concentration at the Extremes (ICE) was implemented as a measurement of spatial social inequality of socioeconomic (Massey, 1996) and racial groups (Krieger et al., 2015, 2016). ICE scores range from −1 to 1 and reflect the extent to which an area's population is concentrated into racial (ICE Race), economic (ICE Income), or combined racial and economic (ICE Race + Income) extremes (Krieger et al., 2015, 2016). Areas with an ICE Race or ICE Income score of −1 indicate that 100% of the population is nonwhite or low‐income, respectively; areas with an ICE Race or ICE Income score of 1 are 100% white or higher‐income, respectively. ICE Race + Income captures the combined effect of racialized economic segregation; an ICE Race + Income score of −1 indicates that 100% of the population is nonwhite and low‐income, while a score of 1 indicates that 100% of the population is white and affluent. ICE has been used as a measurement of structural racism in prior studies assessing the effect of racialized economic inequality on health outcomes (Chambers et al., 2019; Dyer et al., 2022; Krieger et al., 2015; Mitchell et al., 2022; Wallace et al., 2021). To our knowledge, no studies have applied ICE metrics in the context of flood‐related outcomes and disparities in flood risk. ICE scores were calculated for racial, economic, and racialized economic residential inequality at the census tract level using demographic and household income variables from the 2023 ACS in the following equation:

ICE=(A−P)/T,

where *A* represents the population count of the nonwhite and/or low‐income group, *P* represents the population count of the white and/or high‐income group, and *T* represents the total population of the census tract (Krieger et al., 2018; Sugg et al., 2023). For ICE Income, *A* is the number of households with an annual income of less than or equal to $25,000, *P* is the number of households with an annual income of greater than or equal to $125,000, and *T* is the total number of households in each census tract. For ICE Race, *A* is the total number of Black or non‐white Hispanic residents, *P* is the total number of white residents, and *T* is the total population for each census tract. For ICE Income + Race, *A* is the number of Black or non‐white Hispanic residents with an annual income of less than or equal to $25,000, *P* is the number of white residents with an annual income of greater than or equal to $125,000, and *T* is the total number of residents in each census tract. These group definitions and the corresponding formula follow the approach outlined by Krieger et al. (2016). ICE Race, ICE Income, and ICE Race + Income scores at the census tract level are mapped in Figure S2 in Supporting Information S1. ICE index values were multiplied by −1 (inverted) so that higher values indicate greater racial or economic inequality. This transformation aligns the directionality of ICE scores with flood risk for more intuitive interpretation of bivariate LISA results: high‐high clusters represent areas where elevated flood risk spatially coincides with elevated inequality.

### 2.3 Health Data

Data for chronic health conditions at the census tract level in NC were collected from the 2023 release of the Center for Disease Control (CDC)'s PLACES data set (Centers for Disease Control and Prevention, 2023). Chronic conditions that may be further complicated due to flood exposure include diabetes, respiratory disease (e.g., COPD and asthma), kidney disease, and depression (Ratter‐Rieck et al., 2023; Tran et al., 2023). We applied a bivariate analysis of elevated flood risk and elevated prevalence of select chronic health conditions (e.g., asthma, COPD, diabetes, depression, and kidney disease) to identify locations where there is a higher prevalence of underlying populations who may be more susceptible to interruptions in healthcare caused by flood exposure.

### 2.4 RUCA

We calculated the proportion of rural, urban, and suburban census tracts identified in LISA clusters using Rural‐Urban Commuting Area (RUCA) codes (USDA ERS, 2020). RUCA codes classify areas based on population density, urbanization, and commuting patterns. The census tracts were categorized as Urban (RUCA Codes 1–3), Suburban (RUCA Codes 4–7), and Rural (RUCA Codes 8–10). The spatial distribution of RUCA codes are mapped in Figure S3 in Supporting Information S1.

### 2.5 Sensitivity Analyses

We assessed the sensitivity of our results to alternative measurements of economic and racial inequality using the Gini coefficient and the Index of Dissimilarity (IOD), respectively (Austin et al., 2019; Rey & Smith, 2013). The Gini Index and the IOD were derived at the census tract level using values from the 2023 American Community Survey (ACS). We also assessed the consistency of our results at each FloodFactor(™) level comprising the high‐risk category (7, 8, 9, and 10).

## 3 Materials and Methods

### 3.1 Spatial Analysis of Flood Risk and Structural Factors

We computed Global Moran's I as well as univariate and bivariate Local Moran's I statistics. Global Moran's *I* statistics measure spatial autocorrelation among individual variables (univariate) or between two variables (bivariate) across an entire spatial data set. Global statistics can overlook spatial clustering that occurs at the local level. As proposed by Anselin (1995), the Local Moran's *I* statistic can be extended to a bivariate application that assesses the relationship between one variable and the spatial lag of another. This bivariate Local Indicators of Spatial Association (LISA) model can reveal the spatial disparity of the relationship between variables that global measures of spatial autocorrelation might mask (Song et al., 2020). In our analysis, we assessed the relationship between flood risk at each census tract and the spatially lagged values of ICE metrics in neighboring tracts. Thus, high‐high clusters identify census tracts with high flood risk that are surrounded by neighboring tracts with high inequality. We applied bivariate LISA statistics to identify spatially non‐random areas of coincident extremes of flood risk and community‐level indicators. Bivariate LISA maps highlight census tracts with significant (*p* < 0.05) spatial association with their neighboring census tracts. The results of bivariate LISA analysis were categorized into significant groups of positive (e.g., high‐high (HH) or low‐low (LL)) and negative (e.g., high‐low (HL) or low‐high (LH)) spatial autocorrelation among all census tracts in North Carolina. High‐high clusters indicate areas of high flood risk surrounded by high social vulnerability. Low‐low clusters are areas with low flood risks surrounded by low vulnerability. High‐low cluster areas contain high flood risks surrounded by low social vulnerability, while low‐high clusters have low flood risks but high social vulnerability. Tracts not found to have significant spatial association with their neighboring tracts are deemed insignificant. We used the Queen contiguity matrix to define neighbors, which considers all census tracts with a common boundary as a neighbor. Following standard practice, the spatial weights matrix sets diagonal elements to zero (*i* ≠ *j*), excluding each tract from its own neighborhood calculation. LISA significance was assessed using 9,999 permutations, as changing the number of permutations can affect results (Anselin et al., 2014). Observations above the 95% significance threshold were mapped as significant clusters (Anselin, 1995, 2024).LISA analyses were performed in RStudio version 2023.09.1 using the “rgeoda” package and in GeoDa software package version 1.22.0.4 (Anselin, 1995; Anselin et al., 2006).

The exploratory application of both univariate (Koks et al., 2015) and bivariate LISA (Tate et al., 2021) statistics to identify spatial clusters of flood risk and community‐level risk factors can indicate priority communities for resource allocation in NC. Using spatial autocorrelation and LISA methodology emphasizes place‐based patterns of vulnerability and flood risk shaped by underlying structural factors and exacerbated by climate change.

We applied bivariate LISA statistics to identify spatial associations between flood risk and measures of racial residential segregation (ICE Race), economic inequality (ICE Income), and racialized economic segregation (ICE Race + Income) across census tracts and their neighbors. We used census tract‐level data to operationalize the neighborhood level of analysis and capture flood risk patterns at a finer spatial scale. The tract level of analysis exhibits more sensitivity to clustering when compared to larger geographic units (e.g., county). It can provide a more localized understanding of burden distributions (Jones & Kulldorff, 2012).

### 3.2 Secondary Socio‐Demographic Analysis

We used *t*‐tests to compare the socio‐demographic characteristics of tracts identified in HH clusters to state averages to assess the social dimension of community flood risk. Measures included in this comparison were selected based on the Social Vulnerability Index (SoVI) (Cutter et al., 2013; Tate et al., 2021). For each census tract in North Carolina (*n* = 2,672) sociodemographic variables were derived using the 2023 American Community Survey (ACS) with 5‐year estimates from the U.S. Census Bureau. Statistical significance was assumed at alpha = 0.05.

## 4 Results

### 4.1 Global Moran's I

The univariate global Moran's I statistics for economic inequality (ICE Income, 0.568), racial residential segregation (ICE Race, 0.669), racialized economic segregation (ICE Race + Income, 0.565), and high flood risk (0.554) indicate positive spatial autocorrelation, suggesting that areas with similar values tend to cluster within North Carolina (Table S1 in Supporting Information S1).

### 4.2 Univariate Local Moran's I Analysis

**Figure 1:**
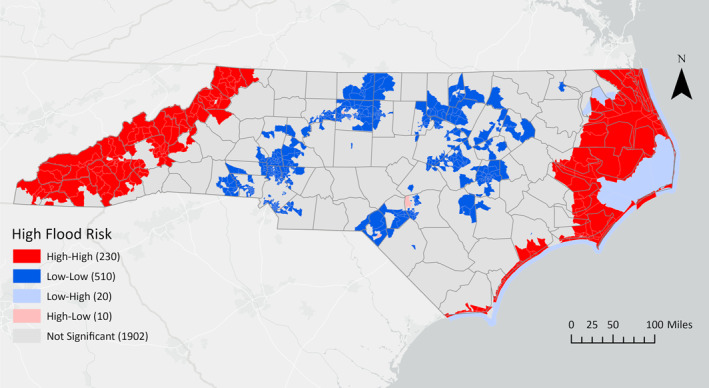
Distribution of high‐high, low‐low, low‐high, and high‐low univariate local indicator of spatial autocorrelation (LISA) clusters for high flood risk at the census tract level in North Carolina.

All HH clusters of flood risk were located within counties situated in the western mountainous and eastern coastal regions of North Carolina (Figure 1).

### 4.3 Bivariate LISA

Bivariate LISA results identified distinct geographic patterns at the census tract level in the spatial clustering of ICE indicators. Table 1 describes the number of census tracts identified in high‐high (HH), low‐low (LL), high‐low (HL), and low‐high (LH) clusters, along with corresponding population counts and density. High–high clusters for ICE Income encompassed the greatest number of census tracts (*n* = 140) and the largest population (*n* = 392,558) compared to clusters identified for ICE Race or ICE Race + Income. In contrast, for ICE Income (tracts = 283, population = 1,385,593) and ICE Race + Income (tracts = 265, population = 1,273,130), low–low clusters were the most prevalent and included the largest populations. For ICE Race, the most common cluster type was high–low (tracts = 280, population = 1,246,766) (Table 1).

**Table 1:** Distribution of Census Tracts Identified in High‐High (HH), Low‐Low (LL), High‐Low (HL), and Low‐High (LH) Clusters of ICE Metrics and Flood Risk

Variable	Cluster type^a^	Tracts in cluster type	Population^b^	Population density^a^ (per mi. sq.)
ICE Income	HH	140	392,558	323.97
	LL	283	1,385,593	1407.45
	LH	110	396,556	444.69
	HL	236	929,036	1525.45
ICE Race	HH	38	74,916	267.59
	LL	240	1,067,853	1167.24
	LH	212	714,198	396.71
	HL	280	1,246,766	1713.22
ICE Race + Income	HH	82	223,523	362.39
	LL	266	1,273,130	1247.40
	LH	168	565,591	384.25
	HL	255	1,041,489	1683.45
All Tracts	N/A	2,672	10,584,340	1374.44

^a^ Clusters and outliers are significant at the *p* < 0.05 level.

^b^ Population values from the 2023 American Community Survey (ACS) with 5‐year estimates.

**Figure 2:**
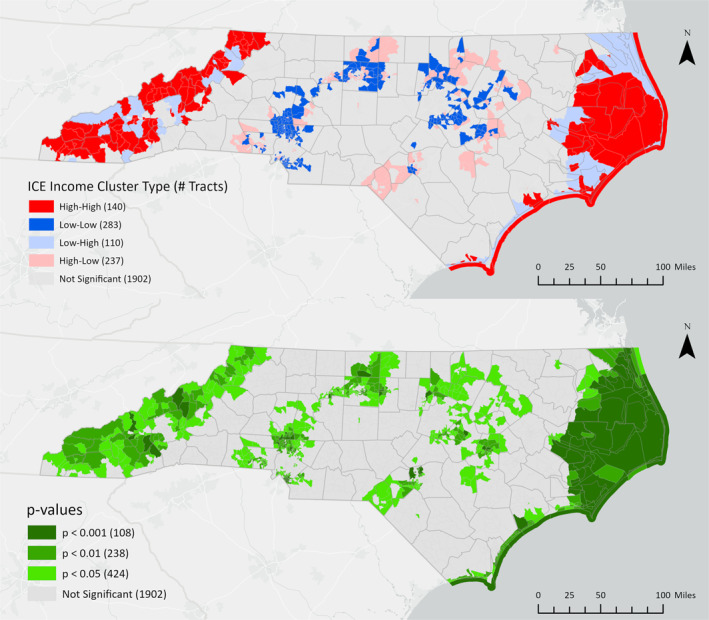
Bivariate LISA clusters and corresponding *p*‐values for flood risk and economic inequality (ICE Income) at the census tract level.

**Figure 3:**
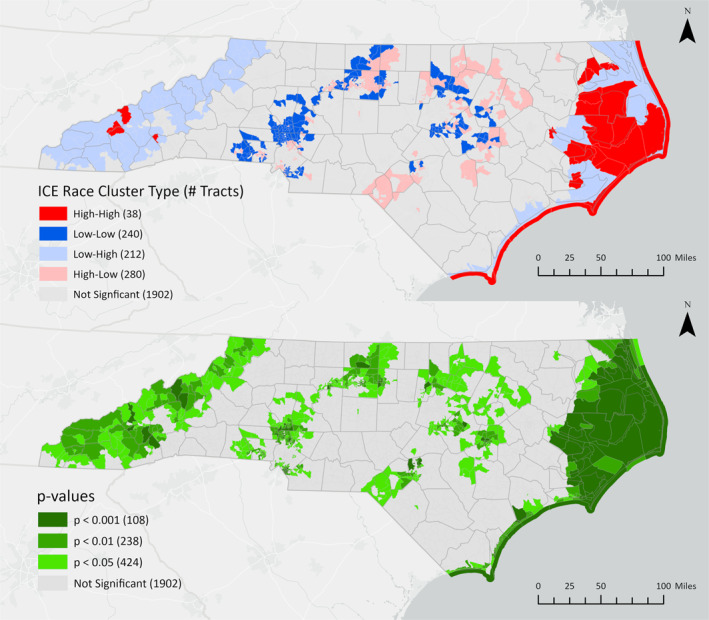
Bivariate LISA clusters and corresponding *p*‐values for flood risk and racial residential segregation (ICE Race) at the census tract level.

**Figure 4:**
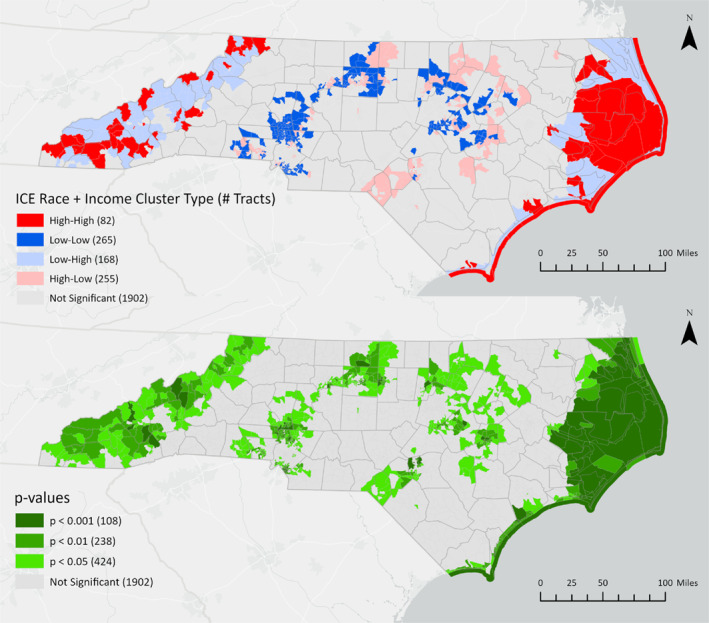
Bivariate LISA clusters and corresponding *p*‐values for flood risk and racialized economic segregation (ICE Race + Income) at the census tract level.

Bivariate LISA cluster locations and their corresponding statistical significance are mapped in Figures 2, 3, 4. Dark red (high‐high) census tracts are regions where areas with high flood risk (measured as the proportion of high‐risk properties) surround areas with high levels of racial (ICE Race), economic (ICE Income), or racialized economic (ICE Race + Income) inequality. Dark blue (low‐low) clusters are areas where low flood risk surrounds low inequality. Light red (high‐low) outliers are locations where high inequality is surrounded by low flood risk, and light blue (low‐high) outliers are locations where low inequality is surrounded by high flood risk. Cluster locations remain consistent at different p‐value thresholds (*p* < 0.05, *p* < 0.01, *p* < 0.001).

All high‐high clusters of flood risk and economic inequality (Figure 2) or racialized economic inequality (Figure 4) were identified in the western and coastal regions of North Carolina (Figure 2). High‐high clusters of racial segregation and high flood risk were predominantly identified in the state's eastern coastal region (Figure 3). Demographic characteristics for high‐high clusters, the percent change compared to all tracts, and statistical significance are shown in Table 2.

**Table 2:** Distinguishing Characteristics and *t*‐Test Results for ICE Metrics and High Flood Risk High‐High Clusters

Dimension	Indicator	ICE income^a^	ICE race^b^	ICE race + income^c^	State average (all census tracts)
Age	Median age (Years)	41.5 (+0.8)	35.1 (−5.6)**	44.6 (+3.9)	40.7
Age	Total population: Less than 5 years old (%)	3.6 (−1.9)***	2.6 (−2.9)***	3.1 (−2.4)***	5.5
Age	Total population: Over 65 years old (%)	35.6 (+18.4)**	22.4 (+5.2)*	31.6 (+14.4)	17.2
Education	No high school (%)	10.2 (−1.3)	8.3 (−3.2)	10.7 (−0.8)	11.5
Employment	Unemployment (%)	4.9 (−0.6)***	4.2 (−1.3)	4.6 (−0.9)	5.5
Health	Population without health insurance (%)	10.8 (−0.1)*	7.8 (−3.1)	10.9 (±0)	10.9
Housing	Occupied housing units: Renters (%)	26.2 (−7.7)***	19.6 (−14.3)**	28.2 (−5.7)*	33.9
Housing	Mobile homes (%)	17.2 (+4.5)***	12.1 (−0.6)	17 (+4.3)**	12.7
Housing	Median housing value for owner‐occupied housing units ($)	204,741 (−9,391)***	100,263 (−113,869)***	162,546 (−51,586)***	214,132
Housing	Families paying more than 30% of income on rent (%)	41.5 (+1.4)*	22.6 (−17.4)***	39.8 (−0.2)	40.0
Income	Median Household Income (in 2023 inflation‐adjusted dollars) ($)	46,708 (−17,752)***	32,769 (−31,691)***	39,920 (−24,540)***	64,460
Dependence	Households receiving social security benefits (%)	39.9 (+6.5)***	27.6 (−5.8)	36.2 (+2.8)	33.4
Race/Ethnicity	White (%)	73.4 (+6.7)**	30.3 (−36.4)***	61 (−5.7)	66.7
Race/Ethnicity	Black (%)	6.9 (−14.3)***	20.9 (−0.3)	11.4 (−9.8)***	21.2
Race/Ethnicity	Asian (%)	0.5 (−2.2)***	0.3 (−2.3)***	0.5 (−2.2)***	2.7
Race/Ethnicity	Hispanic (%)	5.3 (−4.0)***	4.5 (−4.8)***	5.6 (−3.7)***	9.3
Mobility	Total population: No vehicle available (%)	6.4 (+0.5)	5.8 (−0.1)	7.7 (+1.8)	5.9
Internet	No internet access (%)	13 (+0.3)***	9.9 (−2.8)	13.2 (+0.5)**	12.7
Internet	No computer (%)	9.6 (+1.1)***	7.8 (−0.7)	9.5 (+1.0)**	8.5

*Note*. *** = *p* < 0.001, ** = *p* < 0.01, * = *p* < 0.05.

^a^ High flood risk in majority low‐income areas.

^b^ High flood risk in majority non‐white areas.

^c^ High flood risk in majority low‐income and non‐white areas.

High‐high clusters of economic inequality and flood risk are notably distinguished by a significantly greater proportion of residents over age 65 (+18.4%) and white residents (+6.7%), and lower proportions of residents under age 5 (−1.9%), minorities (Black −14.3%; Asian −2.2%; Hispanic −4.0%), and renter households (−7.7%) compared to state averages. These clusters contain a greater proportion of cost‐burdened renter households (+1.4%), mobile home households (+4.5%), households receiving social security (+6.5%), and households without a computer (+1.1%) or internet (+0.3%) access compared to state averages. Median household income (−$17,752) and median housing values (−$9,391) are also lower (Table 2).

Compared to state averages, the population residing within high‐high clusters of racial inequality and flood risk have a significantly lower median age (−5.6 years), a greater proportion of residents above age 65 (+5.2%), and fewer residents younger than age 5 (−2.9%). Within these clusters, there is a smaller proportion of renter households (−14.3%). Median household income (−$31,691), median home values (−$113,869), the proportion of cost‐burdened renters (−17.4%), and the proportion of households receiving social security benefits (−5.8%) are lower within these clusters compared to state averages. These clusters also have a smaller proportion of white (−36.4%), Asian (−2.3%), and Hispanic (−4.8%) residents (Table 2).

High‐high clusters of racialized economic segregation and flood risk are distinguished by fewer residents under age 5 (−2.4%), fewer renters (−5.7%), more mobile homes (+4.3%), lower median housing value (−$51,586), lower median household income (−24,540), fewer minorities (Black −9.8%, Asian −2.2%, Hispanic −3.7%), and more households without computer (+1.0%) or internet (+0.5%) access (Table 2).

Census tracts that were identified in high‐high clusters for economic inequality, racial segregation, or racialized economic inequality are primarily urban (57.8%, 44.7%, and 50.0%, respectively) (Table 3). A greater proportion of rural census tracts were identified in HH clusters for racial segregation (34.2%) and racialized economic segregation (31.2%) compared to economic inequality (25.7%). Suburban tracts comprised 16.4% of HH clusters for ICE Income, 21.0% for ICE Race, and 20.7% for ICE Race + Income. RUCA distributions for LL, HL, and LH clusters are presented in Table 3.

**Table 3:** Rural‐Urban Commuting Area (RUCA) Code Distribution for High‐High, Low‐Low, High‐Low, and Low‐High Clusters of Racial Segregation (ICE Race), Economic Inequality (ICE Income), and Racialized Economic Inequality (ICE Race & Income) and Flood Risk

	Rural (%)	Suburban (%)	Urban (%)
High‐high Clusters			
ICE Income: Majority low‐income	36 (25.7)	23 (16.4)	81 (57.8)
ICE Race: Majority nonwhite	13 (34.2)	8 (21.0)	17 (44.7)
ICE Race + Income	25 (30.5)	16 (19.5)	41 (50.0)
Low‐low Clusters			
ICE Income: Majority high‐income	1 (0.3)	5 (1.7)	277 (97.8)
ICE Race: Majority white	2 (0.8)	11 (4.5)	227 (94.5)
ICE Race + Income	1 (0.3)	6 (2.2)	259 (97.7)
Low‐high Clusters			
ICE Income	9 (8.1)	23 (20.9)	78 (70.9)
ICE Race	32 (15.0)	38 (17.9)	142 (66.9)
ICE Race + Income	20 (11.9)	30 (17.8)	118 (70.2)
High‐low Clusters			
ICE Income	6 (2.5)	23 (9.7)	207 (87.5)
ICE Race	5 (1.7)	18 (6.4)	257 (91.7)
ICE Race + Income	6 (2.3)	24 (9.4)	225 (88.2)

**Figure 5:**
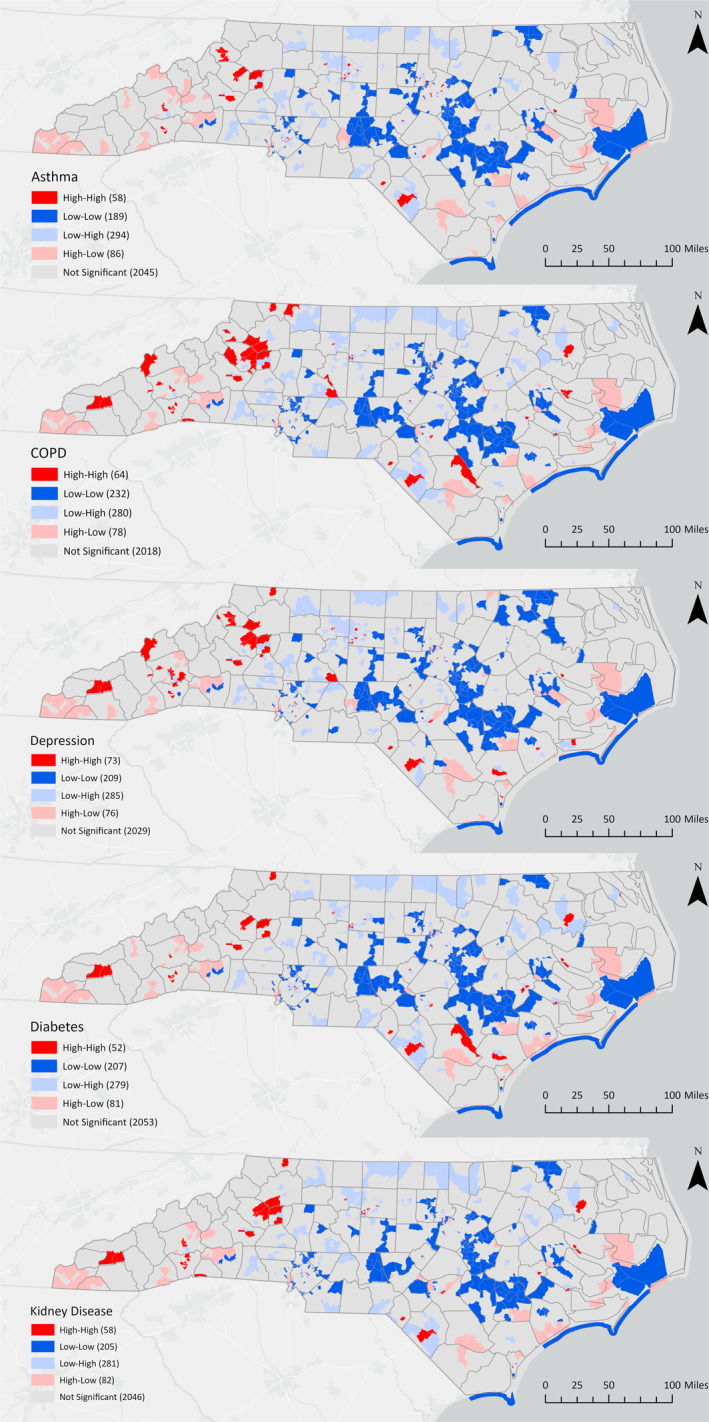
Bivariate LISA clusters and corresponding *p*‐values for flood risk and asthma, COPD, depression, diabetes, and kidney disease at the census tract level.

Additional bivariate LISA analysis of flood risk and health outcomes identified areas with elevated flood risk and high prevalences of asthma, COPD, depression, diabetes, and chronic kidney disease (Figure 5). High‐high clusters for health outcomes emerged in counties located in the western mountainous and southeastern coastal regions of North Carolina.

Figures S5 and S6 in Supporting Information S1 map bivariate LISA clusters with alternative measures of inequality (Gini coefficient and Index of Dissimilarity). High‐high clusters for both metrics are present in the eastern and coastal regions of the state. Figures S7 to S10 in Supporting Information S1 present supplemental analysis of alternative flood risk levels (FF7, FF8, FF9, FF10). Results for FF10 and FF9 were consistent with the main findings, while there were fewer clusters in the western region at FF8 and FF7. We selected these categories because they comprised the high‐risk category (FF ≥ 7) and were representative of severe (9–10) or extreme (7–8) risk categories as defined by the FSF‐FM.

## 5 Discussion

Our analysis identifies regions in North Carolina where flood risk converges with socioeconomic inequality, racial residential segregation, and elevated health vulnerabilities, highlighting the spatial relationship between community structural factors and population exposure to flood hazard. This study focuses on the spatial distribution of high–high clusters, as these areas experience the compounded burden of flood risk and community risk factors. Structural inequities such as lower housing values, a higher prevalence of mobile homes, greater dependence on social security income, and lower median household incomes amplify vulnerabilities in areas identified in HH clusters. Access to essential resources, including healthcare, transportation, and internet connectivity, is also significantly lower in these communities.

Our identification of clusters of high flood risk and racialized economic inequality in the eastern region of the state aligns with prior studies documenting coastal flood risk in North Carolina (Alipour et al., 2020; de Vries, 2011; Pricope et al., 2019; Thomson et al., 2021). Select coastal areas in the southeastern region were also identified as high‐high clusters with a prevalence of chronic health conditions. These findings affirm well‐documented vulnerabilities in coastal regions. Coastal high‐high clusters, in contrast, are distinguished by larger Black populations and similarly marked by economic disparities. Across both regions, these clusters represent areas where economic inequality and flood risk intersect to exacerbate community vulnerability.

The incorporation of both inland and coastal flood metrics into our analysis also reveals significant clusters of flood risk, economic inequality, and chronic health conditions (e.g., asthma, COPD, depression, diabetes, and kidney disease) in the western mountainous region of NC, a region that has historically been underrepresented in flood risk research (Wang & Sebastian, 2021). These findings contrast with FEMA's special flood risk area maps, which guide mandatory flood insurance for federally backed mortgages, leaving many homeowners impacted by Tropical Storm Helene without coverage and unprepared for recovery costs. Preliminary estimates using First Street Foundation data suggest that the number of at‐risk properties in western North Carolina could be up to seven times higher than FEMA maps indicate (Crowe et al., 2024). The unprecedented flooding from Hurricane Helene in September 2024 exposed vulnerabilities in communities identified in high–high clusters. Demographically, western high‐high clusters are home to larger proportions of young children (<5 years old), older adults (>65 years old), and white populations compared to other tracts in North Carolina.

While most clusters are urban, the presence of high–high clustering in rural areas demonstrates the interaction between flood risk and socioeconomic inequality in these settings, consistent with prior research on rural vulnerability to flooding and structural inequities (Rhubart & Sun, 2021). Helene caused an estimated $59.6 billion in damages, primarily in rural Appalachian areas, with major impacts to transportation and other infrastructure, despite lower population density than prior hurricanes such as Florence (2018) ($16.7 billion in damage) (NWS, 2018; OSBM, 2024). These findings demonstrate the unequal burden of flood disasters on vulnerable populations and emphasize the urgent need for targeted interventions in high–high cluster areas. By prioritizing these regions, local and state authorities can reduce disaster risks, enhance climate adaptation strategies, and support equitable recovery efforts.

### 5.1 Implications

Our analysis highlights NC's western mountainous and eastern coastal regions as areas with elevated flood risk and strong associations with structural racism, income inequality, racialized economic disparities, and health vulnerabilities. While our statistics are not explanatory, high‐high clusters from the bivariate LISA analysis identify priority areas where flooding coincides with other community risk factors, guiding resource allocation at state and local levels. High‐low and low‐high clusters also indicate areas with either elevated flood risk or neighborhood inequality. By examining chronic conditions alongside flood risk, we identified regions where public health resources should be prioritized before, during, and after flooding, as these populations are more vulnerable to climate exposures and related health impacts, illustrated recently by the unprecedented flooding from Tropical Storm Helene in western NC. Recognizing areas with high flood risk, significant disparities, and sensitive health conditions is essential for targeting climate adaptation efforts. Programs such as the North Carolina Department of Health and Human Services Climate and Health Program aim to enhance resilience in communities facing high exposure, elevated sensitivity, and low adaptive capacity.

### 5.2 Strengths and Limitations

This study has several strengths. We employed an exploratory spatial analysis approach to identify community‐level drivers associated with elevated flood risk, economic inequality, structural racism, and chronic health conditions, enabling the identification of priority areas for targeted interventions and resource allocation. Using census tracts provides a useful proxy for neighborhood‐level analysis of community characteristics.

There are also limitations. Flood risk estimates from the First Street Foundation Flood Model may overestimate risk in some areas due to unaccounted local protections, and the census tract scale may mask finer‐scale vulnerability. Bivariate LISA analyses are subject to multiple comparisons, potentially producing false positives, which we mitigated by assessing cluster significance at conservative thresholds (*p* < 0.05, 0.01, 0.001). LISA results indicate spatial associations but not causal relationships. Results may also be sensitive to neighborhood weight definitions and boundary effects. Future work could expand to multistate or national analyses and normalize flood risk by buildable land density.

## 6 Conclusions

This study highlights the association between racial and economic factors and the geographic distribution of flood risk burden. Results suggest that flood risk overburdens specific racial and economic communities in certain geographic regions across North Carolina and emphasize the role of place‐based factors in understanding flood risks and determining priority areas for allocating financial and structural resources. Financial resources include disaster relief funds and grants for flood mitigation infrastructure that can be directed toward vulnerable areas identified in this study in anticipation of increased flood events and in the aftermath of flood exposure. Our analysis identified areas where high flood risk is clustered with economic inequality, structural racism, and a high prevalence of chronic illness; these areas are concentrated in the western and coastal regions of North Carolina. This study draws attention to flood risk in the western region of the state, an area often overlooked in traditional flood risk analyses. This analysis can be extended to public health practice by integrating flood risk data and health condition data to identify areas for targeted public health interventions during and after flood events. Potential public health initiatives include providing emergency preparedness training to residents in high‐risk zones, establishing mobile health clinics for post‐flood care, and developing early warning systems that consider the specific vulnerabilities of each community.

## Conflict of Interest

The authors declare no conflicts of interest relevant to this study.

## Supporting information

Supporting Information S1

## Data Availability

The Flood Model (FSF‐FM) data set used to measure flood risk in the study is available from the First Street Foundation (First Street Foundation, 2021). Version 1.22.0.04 of GeoDa is free and open source (Anselin, 2025).
