# Supplementary material for: Spatial Analysis of Flood Risk, Neighborhood Characteristics, and Chronic Health Conditions in North Carolina
Source: Geohealth. 2026 Feb 2;10(2):e2024GH001295. doi: 10.1029/2024GH001295 (PMC12862280; doi:10.1029/2024GH001295)
Supplement: Supplementary file 1 — Supporting Information S1 [file GH2-10-e2024GH001295-s001.docx]

*GeoHealth*

Supporting Information for

**Spatial analysis of flood risk, neighborhood characteristics, and chronic health conditions in North Carolina**

**Authors: S.E. Ulrich^1^, M.M. Sugg^1^, S.M. Hatcher^2^, and J.D. Runkle^3^**

^1^Department of Geography and Planning, P.O. Box 32066, Appalachian State University, Boone, NC 28608, contact email: ulrichse@appstate.edu

^2^Occupational and Environmental Epidemiology Branch, Division of Public Health, North Carolina Department of Health and Human Services, 5505 Six Forks Rd, Raleigh, NC 27609

^3^North Carolina Institute for Climate Studies, North Carolina State University, 151 Patton Avenue, Asheville, NC 28801

Corresponding author: Sarah E. Ulrich ([ulrichse@appstate.edu)](about:blank)

**Contents**

Figure S1…………………………………………………………………………………………………………………………2

Figure S2…………………………………………………………………………………………………………………………3

Figure S3…………………………………………………………………………………………………………………………4

Figure S4…………………………………………………………………………………………………………………………5

Figure S5…………………………………………………………………………………………………………………………6

Figure S6…………………………………………………………………………………………………………………………7

Figure S7…………………………………………………………………………………………………………………………8

Figure S8…………………………………………………………………………………………………………………………9

Figure S9………………………..…………………………………………………………………………………………….10

Figure S10……………………..………………………………………………………………………….………………….11

Table S1…………………………………………………………………………………………………….………………….12

Table S2…………………………………………………………………………………………………….………………….13

Table S3………………………………………………………………………………………………………………………..14

**
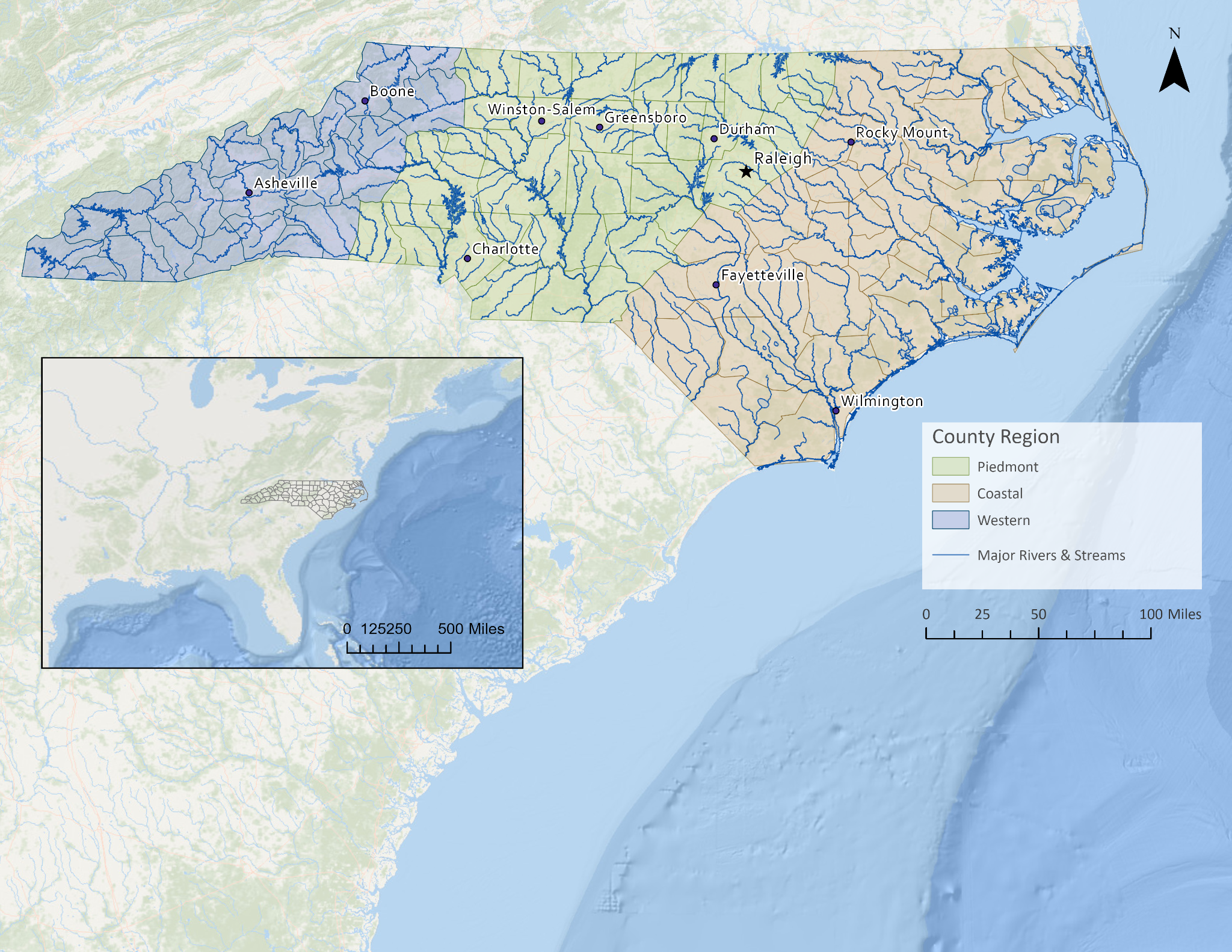
**

**Figure S1.** North Carolina’s physiographic regions, county boundaries, major cities, and major rivers.

**
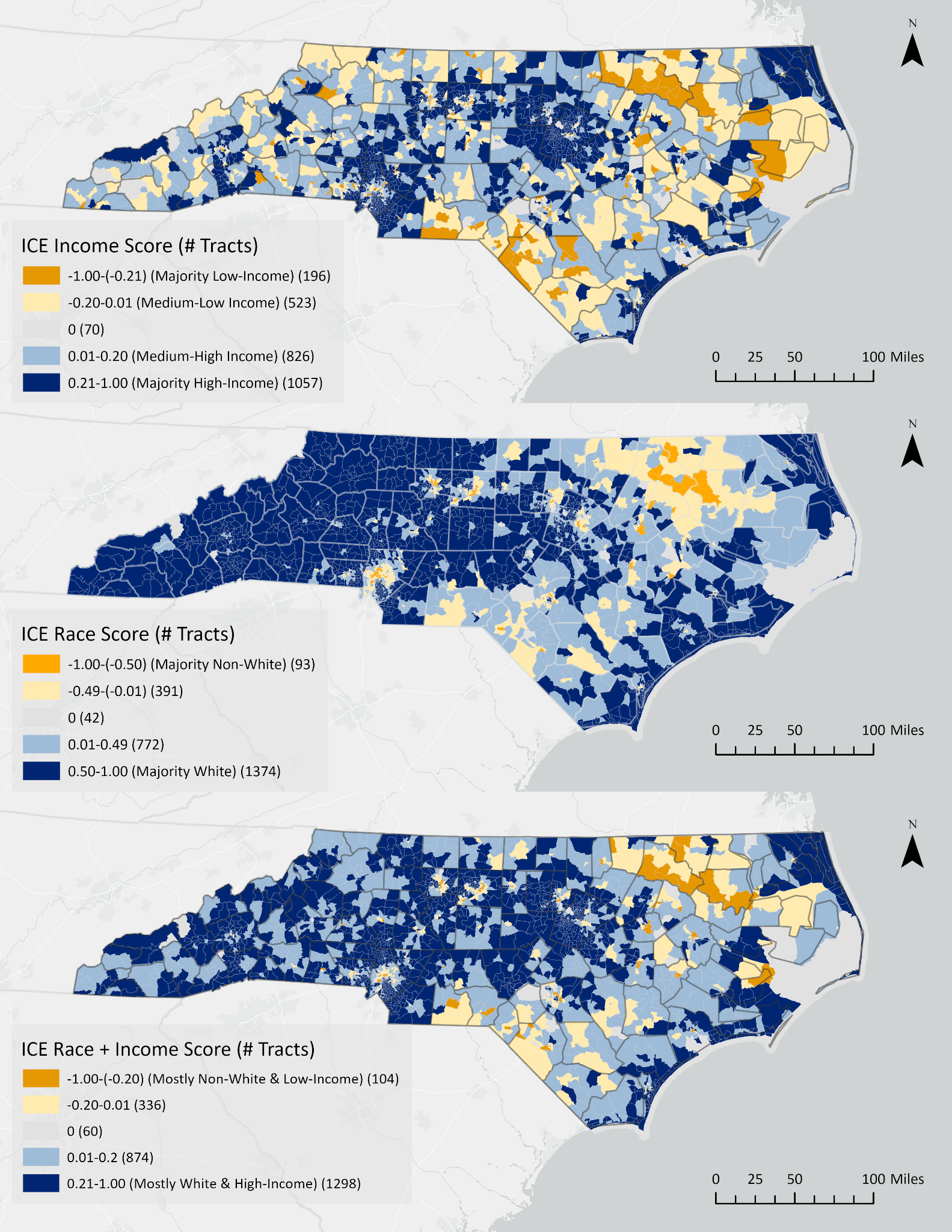
**

**Figure S2.** Distribution of ICE Race, ICE Income, and ICE Race + Income scores at the census tract level in North Carolina, derived using values from the 2023 ACS with 5-year estimates


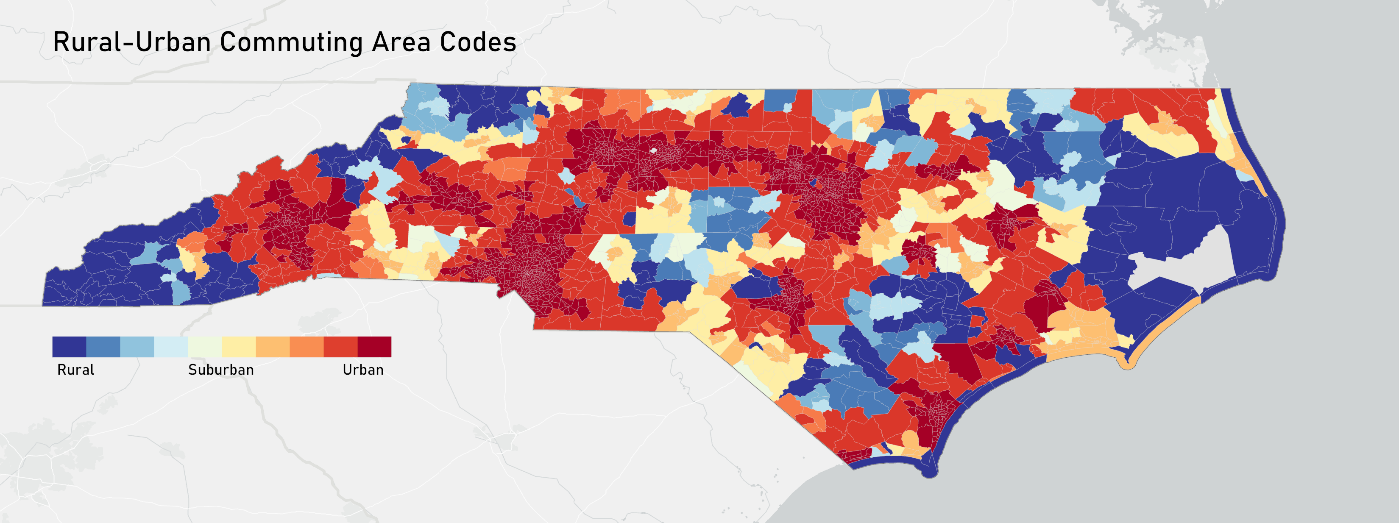


**Figure S3.** Distribution of Rural-Urban Commuting Area (RUCA) codes at the zip code tabulation area (ZCTA) level in North Carolina


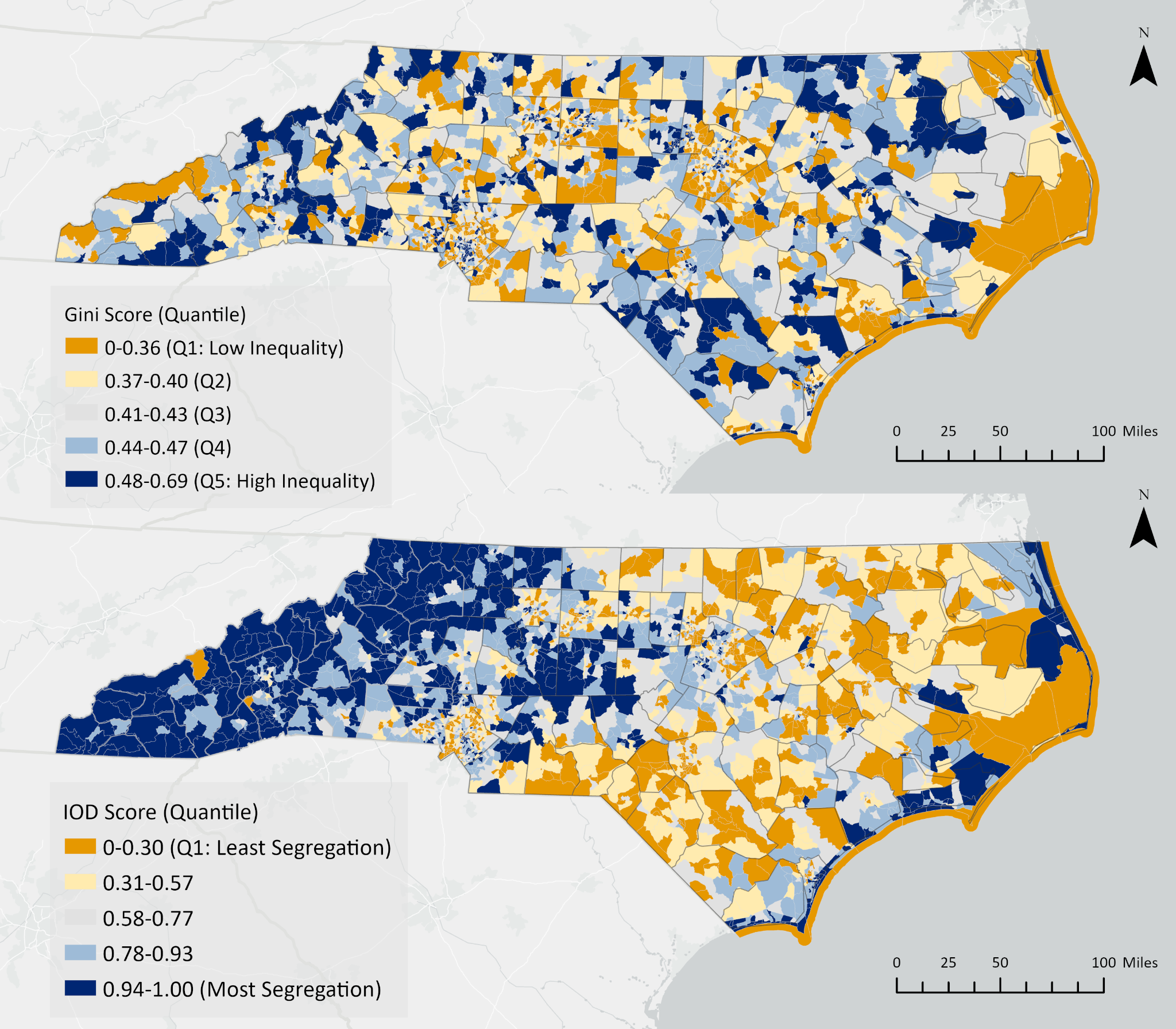


**Figure S4.** Distribution of Gini and Index of Dissimilarity (IOD) quantiles at the census tract level


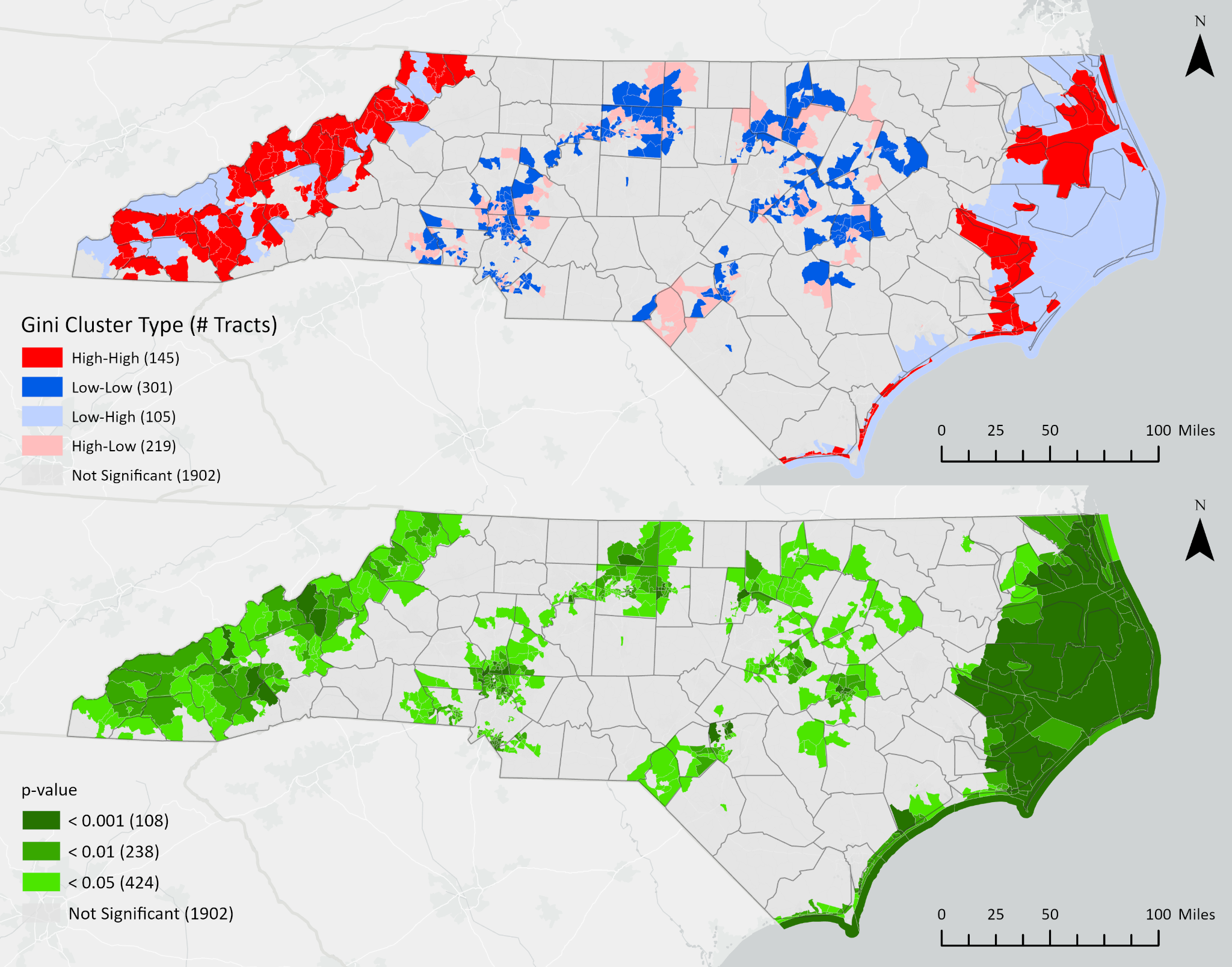


**Figure S5.** Bivariate LISA clusters and corresponding p-values for flood hazard and the Gini Index of Inequality at the census tract level

**
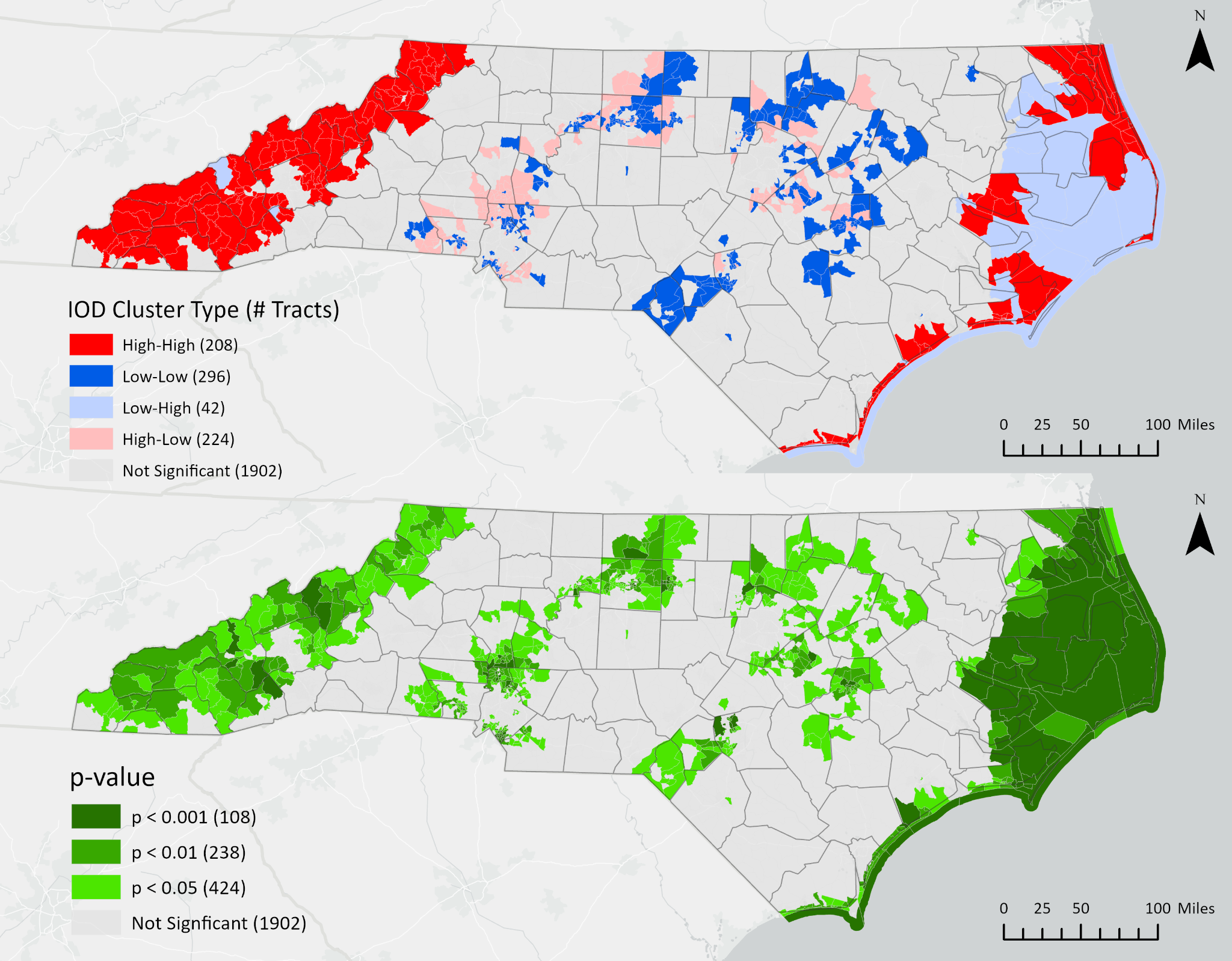
**

**Figure S6.** Bivariate LISA clusters and corresponding p-values for flood hazard and the Index of Dissimilarity (IOD) at the census tract level.


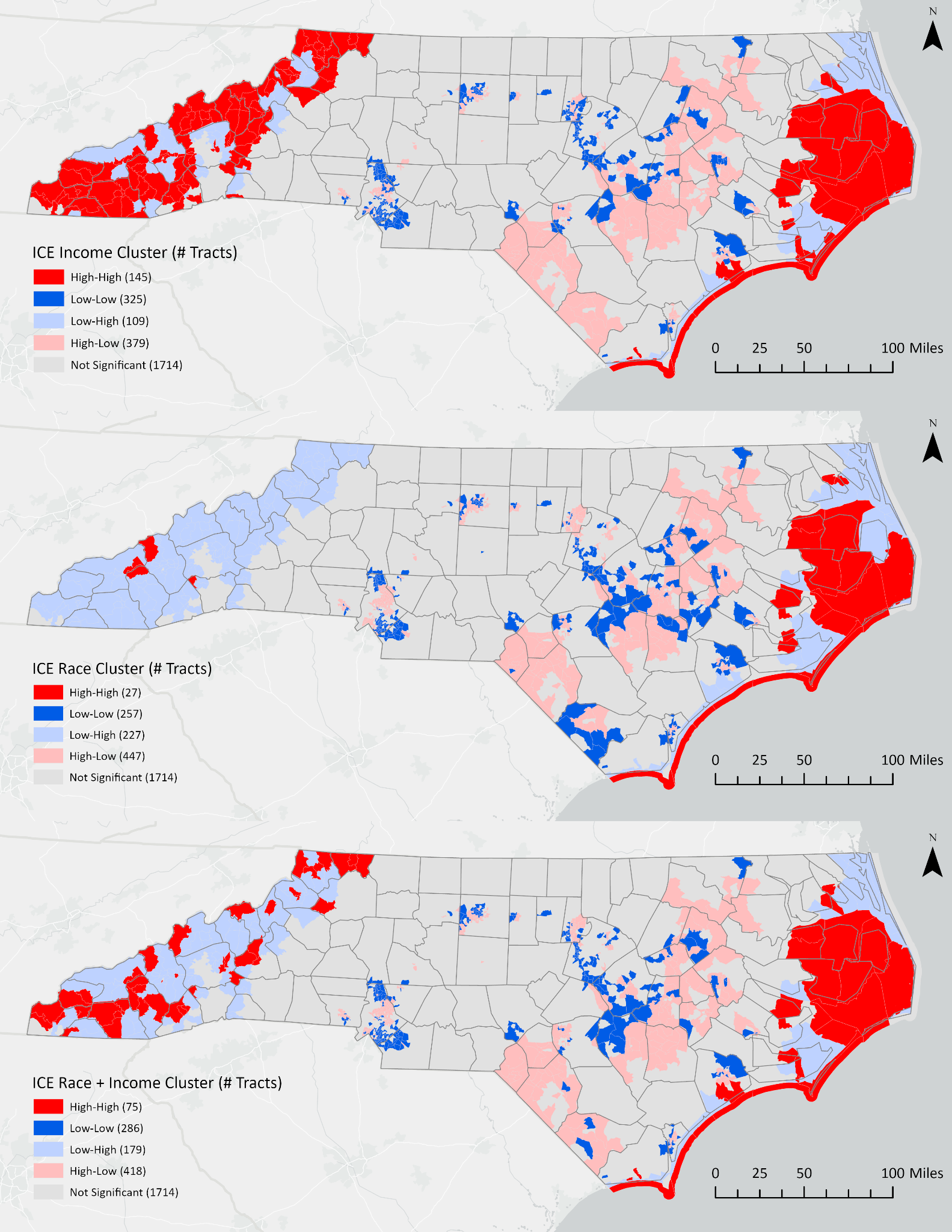


**Figure S7.** Bivariate LISA clusters of ICE metrics and the percent of properties in each census tract with a FloodFactor(™) score of 10


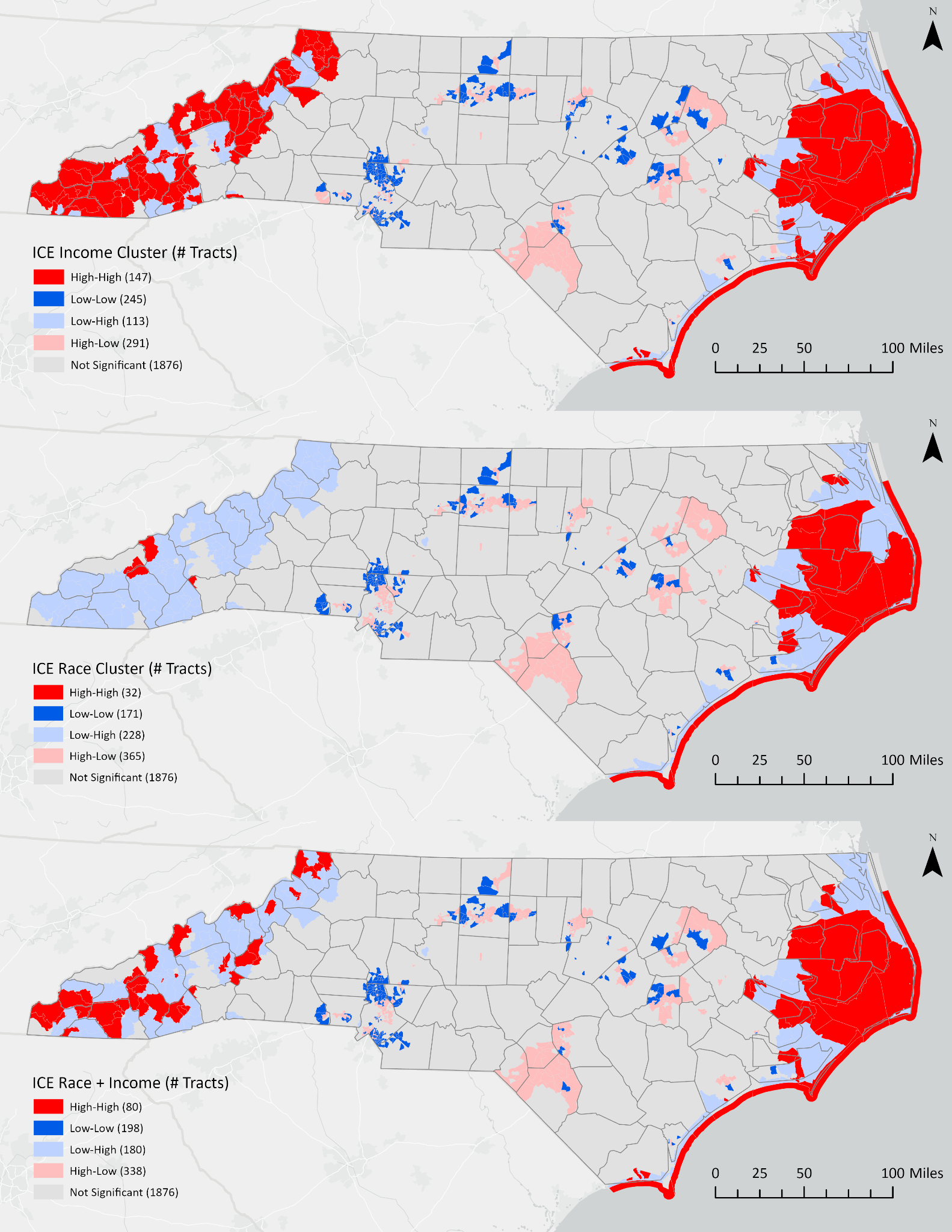


**Figure S8.** Bivariate LISA clusters of ICE metrics and the percent of properties in each census tract with a FloodFactor(™) score of 9


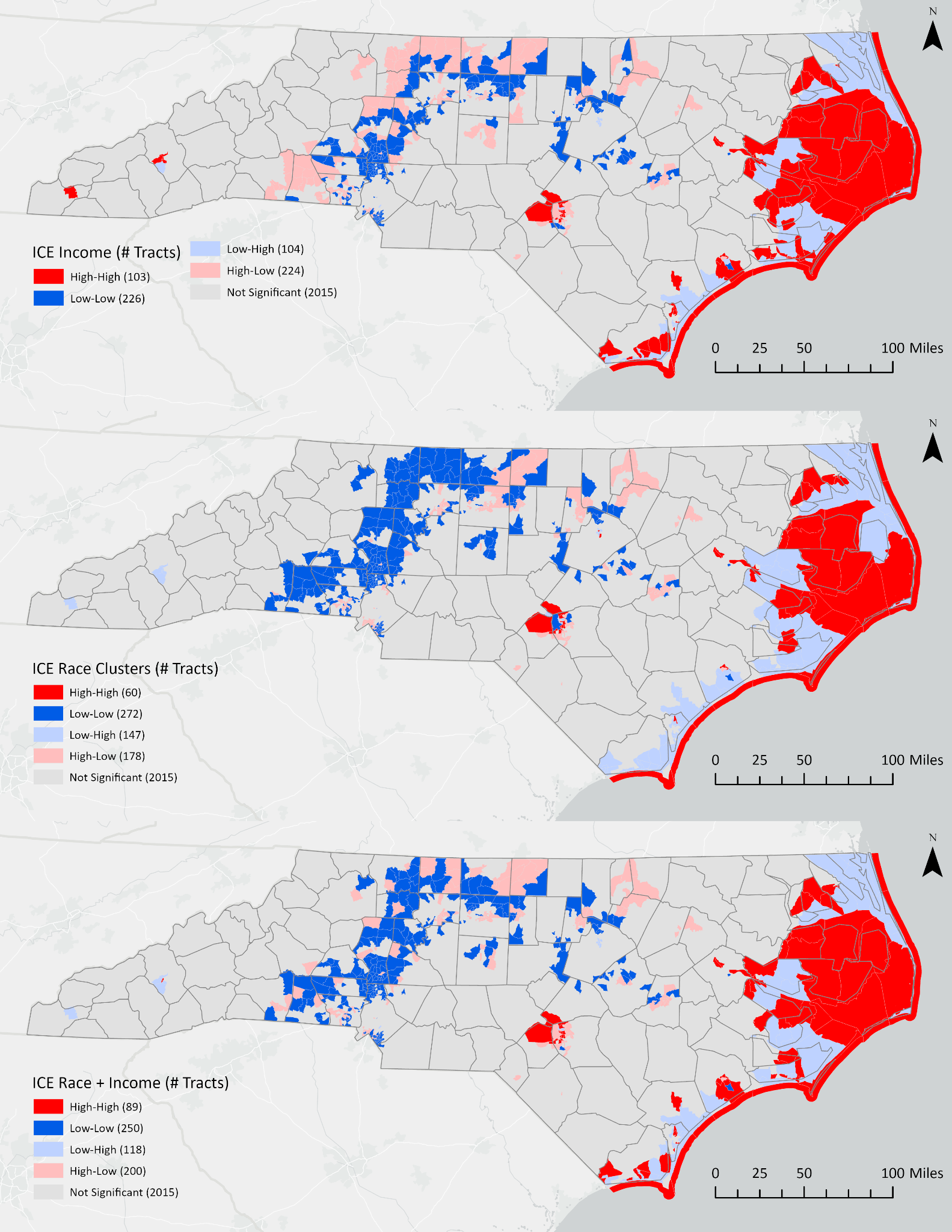


**Figure S9.** Bivariate LISA clusters of ICE metrics and the percent of properties in each census tract with a FloodFactor(™) score of 8


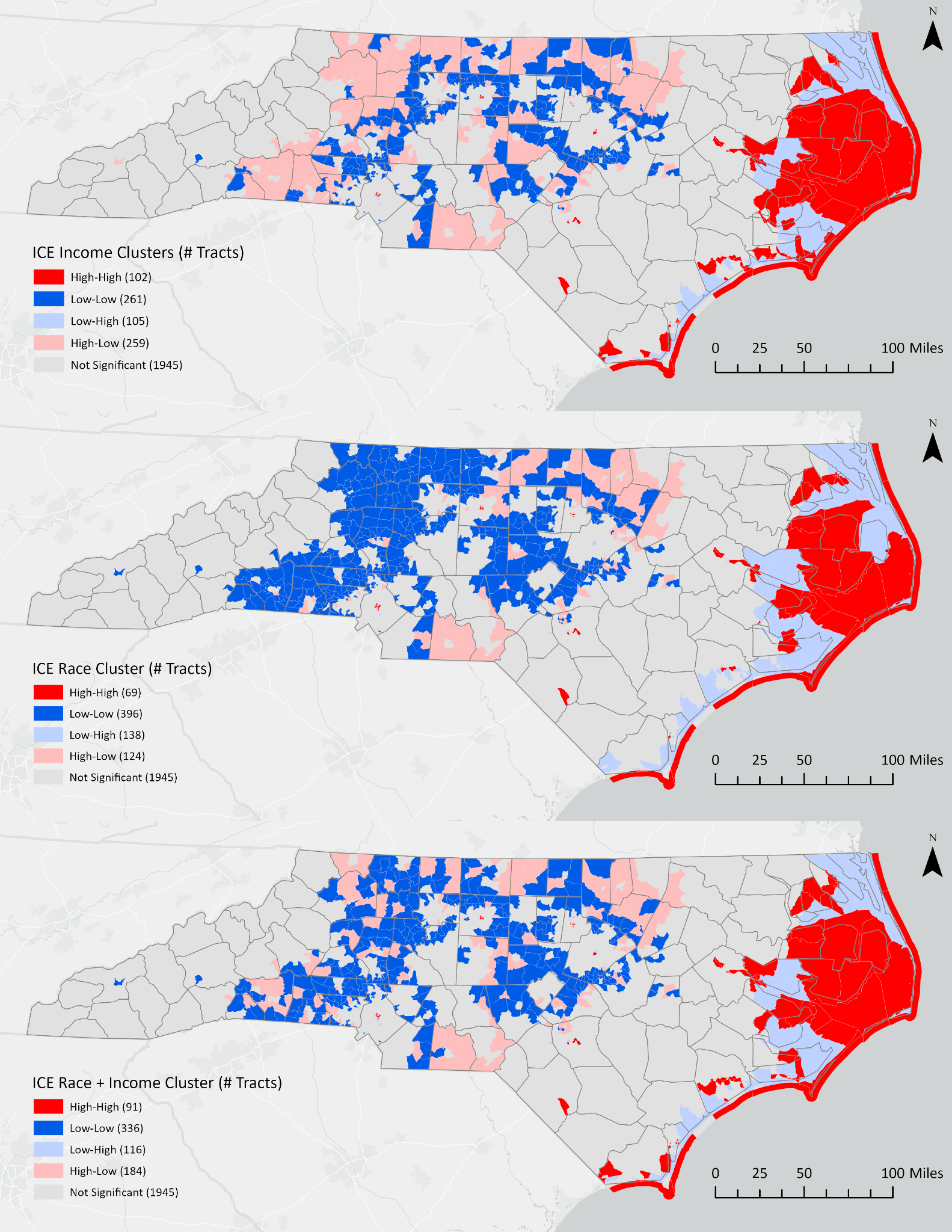


**Figure S10.** Bivariate LISA clusters of ICE metrics and the percent of properties in each census tract with a FloodFactor(™) score of 7

| **Variable** | **Global Moran’s *I*** |
| --- | --- |
| High flood risk | 0.554 |
| ICE Income | 0.568 |
| ICE Race | 0.669 |
| ICE Race + Income | 0.565 |
| Gini | 0.196 |
| Index of Dissimilarity | 0.498 |

**Table S1.** Global Moran’s I values for flood risk and measurements of inequality
